# Relative resistance of *Salmonella* serotypes (Typhimurium, Infantis, and Reading) to peroxyacetic acid on chicken wings

**DOI:** 10.1016/j.psj.2024.103935

**Published:** 2024-06-01

**Authors:** S. Vaddu, J. Wang, G. Sidhu, C. Leone, M. Singh, H. Thippareddi

**Affiliations:** ⁎ Department of Poultry Science, University of Georgia, Athens, GA, 30602, USA; † Department of Food Science and Technology, University of Georgia, Athens, GA 30602, USA

**Keywords:** peroxyacetic acid (PAA), *salmonella*, serotype, chicken wing, decomposition

## Abstract

Peroxyacetic acid (**PAA**) is widely used as an antimicrobial in poultry processing. Recent salmonellosis outbreaks caused by *Salmonella* Infantis (**SI**) from chicken products and *Salmonella* Reading (**SR**) from turkey products have raised concerns about their enhanced resistance (compared to *Salmonella* Typhimurium [**ST**]) to commonly used antimicrobial interventions such as PAA. The objective of this research was to evaluate the efficacy of PAA against *Salmonella* serotypes (Typhimurium, Infantis and Reading), effect on product color and decomposition of PAA at different pH levels. Fresh chicken wings (0.45 kg) were inoculated with a cocktail (ca. 6 log CFU/mL) of nalidixic acid resistant ST, rifampicin resistant SI and kanamycin resistant SR. Inoculated chicken wings were immersed in PAA solutions (100 or 500 ppm; adjusted to either pH 8.5 or unadjusted natural pH) for either 10 s or 60 min to replicate treatments for chicken parts or whole carcasses, respectively. Treated chicken wings were rinsed in buffered peptone water (100 mL) containing sodium thiosulfate (0.1 %), serially diluted in peptone water supplemented with 200 ppm of nalidixic acid, rifampicin or kanamycin for enumeration of ST, SI, and SR respectively, and plated on APC Petrifilm. Immersion of chicken wings in 500 ppm PAA for 60 min resulted in greater microbial reductions (*P* ≤ 0.05) of ST, SI, SR of ca. Two log CFU/mL each, compared to 10 s treatment. Regardless of concentration and pH of PAA, increased exposure time (60 min vs. 10 s) resulted in greater reductions (*P* ≤ 0.05) of ST, SI, SR. ST was slightly more resistant to PAA solutions than *S*. Infantis and *S*. Reading (*P* ≤ 0.05) for all experimental conditions (PAA conc, pH, and exposure times). Faster decomposition of PAA (100 and 500 ppm) was observed at pH 8.5 compared to unadjusted, natural pH (*P* ≤ 0.05). Product color (lightness, L*) was not affected regardless of the PAA concentration, exposure time or the pH.

## INTRODUCTION

Poultry is the most consumed meat type in the United States, and outbreaks of *Salmonella* due to consumption of undercooked or contaminated poultry products continue to remain a high risk globally (USDA-FSIS, 2016). *Salmonella enterica* subsp. *enterica* continues to be a leading cause of salmonellosis, which is a major foodborne illness responsible for an estimated 1.2 million human illnesses with more than 23,000 hospitalizations and 450 deaths in the United States each year (Scallan et al., 2011). Salmonellosis can lead to gastroenteritis mainly caused by non-typhoidal serovars clinically manifested by nausea, vomiting and profuse diarrhea (Als et al., 2018). Although a variety of sources can contribute to salmonellosis, animals including poultry, pigs, rodents, cattle and many others serve as principal reservoirs, that can lead to human infections (Chlebicz & Śliżewska., 2018). Poultry meat and eggs are predominant reservoirs of *Salmonella* in the United States (Cosby et al., 2015).

From 2006 to 2020, more than 153 *Salmonella* multistate foodborne disease outbreaks have been reported in the United States, affecting more than 16,849 human illnesses (Jingxuan et al., 2021). Poultry meat and eggs were responsible for over 19% of the outbreaks (Jingxuan et al., 2021). According to *Salmonella* Annual Report (2016), majority of serotypes reported to cause *Salmonella* infections include Enteritidis, Newport, Typhimurium, Javiana, I4, [5],12: i: -, and Infantis. A large multi-state *Salmonella* Infantis (**SI**) outbreak between 2018 and 2019 caused 129 human illnesses and the Centers for Disease Control and Prevention (**CDC**) investigation indicated that the outbreak strain was from raw chicken products contaminated by SI (Basler et al., 2019). Moreover, incidence of *S.* Infantis recorded the largest proportion increase by >165.8% between 2006 and 2016 across the United States. Similarly, *S.* Reading is a serotype that was not commonly associated with human illness, but a large multistate outbreak of 358 *S*. Reading illnesses occurred between 2018–2019. The CDC, the U.S. Department of Agriculture (**USDA**), and the U.S. Food and Drug Administration (**FDA**) reported that the outbreak strain of *S*. Reading was associated with contaminated turkey products (CDC, 2019). The decrease in prevalence of *S*. Typhimurium and *S*. Enteritidis and large proportional increase in the *S*. Infantis and *S*. Reading human illnesses in the past decade suggests an emerging problem for the poultry industry. This could possibly be due to the increased use of *S*. Typhimurium and *S*. Enteritidis vaccines at the breeder level and lack of vaccine cross-protection between different serovars of poultry. It is speculated that this caused other serovars to occupy the poultry gut niche, resulting in human illnesses from the newer serovars such as *S*. Infantis and *S*. Reading. Recent salmonellosis outbreaks caused by *S.* Infantis from chicken products and *S.* Reading in turkey products have raised concerns among poultry processors about their enhanced resistance to commonly used antimicrobial interventions in poultry processing.

*Salmonella* can be introduced into poultry production system at either pre-harvest and/or post-harvest stages from multiple sources. Birds get infected either by vertical transmission through chicks hatching from the contaminated eggs or by horizontal transmission through contaminated feed, water, litter, wild birds, rodents, beetles and disseminate *Salmonella* spp. on the farm through the oral-fecal route (Nair & Johny, 2019). Chickens can naturally harbor foodborne pathogens on their skin, feathers, gastro-intestinal tract and feet which can be transported to poultry processing plant. Further, damage to the gastro intestinal tract during the evisceration process can lead to carcass contamination and transfer to other carcasses (Singh et al., 2019). Further, Cason et al. (2007) also reported the prevalence of external or internal foodborne pathogens on broilers prior to processing, with *Salmonella* levels of up to 2.0, 3.1, and 2.6 log CFU/g on feathers, in crop, and ceca of broilers, respectively. These high initial loads of microbial populations pose a challenge for poultry processors in reducing and/or eliminating *Salmonella* in poultry meat. Poultry processing industry relies heavily on antimicrobial interventions to reduce prevalence and concentrations of foodborne pathogens *Salmonella* and *Campylobacter*. Peroxyacetic acid (**PAA**) is widely used during poultry processing as an antimicrobial and is an equilibrium mixture of acetic acid, hydrogen peroxide, and water (Bauermeister et al., 2008). Peroxyacetic acid degrades to acetic acid, oxygen, and water without any toxic residues. This makes it superior compared to most other poultry-processing antimicrobials such as sodium chlorite and other chlorine-based antimicrobials which release trihalomethanes in the presence of organic matter (Micciche et al., 2018). Some trihalomethanes such as chloroform and bromodichloromethane are considered potential human carcinogens and can contribute to occupational hazard exposure to plant workers (Cressey et al., 2008). Further, chlorine can cause bleaching of the broiler skin during processing, especially when used at higher concentrations (Bauermeister et al., 2008). Utilization of PAA has increased significantly among poultry processors and currently, majority of poultry processors have switched from use of chlorine in their chillers and other antimicrobial interventions to PAA. The mechanism of action of PAA solutions against bacteria could be either from the oxidizing mechanism of PAA and H_2_O_2_ or due to the weak acid mechanism of acetic acid (Vaddu et al., 2021a). Peroxyacetic acid has been extensively used as an antimicrobial in chillers as well as a post-chill dip and/or in finishing chillers (Kumar et al., 2020). PAA is approved for use by the United States Department of Agricultures’ Food Safety and Inspection Service (USDA-FSIS) as “generally recognized as safe” (USDA-FSIS, 2020, Olson et al., 2020). PAA can be either obtained in concentrated form and stored under nonhazardous conditions and diluted for use or generated on-site (Vaddu et al., 2021b).

Most poultry processors prefer to adjust the PAA solutions to alkaline range to prevent loss of moisture due to extended exposure to low (natural) pH of PAA solutions. Since PAA disassociates at pH values greater than the pKa (8.2), it is important to evaluate the effect of PAA solution pH on decomposition rate and the effective concentration over the exposure time.

Color is one of the major quality attributes that can affect consumer perception and thus purchasing decisions (Fletcher, 1992). Color of broiler skin, although not a true indicator for bird's health status, a more “golden yellow” color can be indicative of healthy birds in some regions of the United States (Bauermeister, 2008). Consumers’ acceptance and buying behavior plays a major role in the commercial success of poultry production for poultry marketed as whole birds or for parts that have skin. Previous studies reported a visible color change on the skin of chicken carcasses at PAA concentrations >200 ppm, although not statistically significant (Bauermeister, 2008; Chen et al., 2014). The lighter poultry skin color after treating with PAA could be due to its oxidizing properties (Chen et al., 2014). Yang and Chen (1993) reported a positive correlation between pH and color of ground chicken meat. As pH increases, the lightness (L*) and yellowness (b*) values decreased, but redness (a*) increased. However, such correlation was not observed in other studies (Chen et al., 2014; Laranja et al., 2021), probably due to the natural variability in chicken meat or skin.

Comparative studies on efficacy of PAA on different *Salmonella* serovars has not been reported. Thus, the objectives of this study were to evaluate the effect of PAA exposure (with varying concentrations, pH and exposure times) on 1) reduction in populations of 3 serotypes of *Salmonella* (Typhimurium, Reading and Infantis), 2) skin color and moisture gain of chicken wings, and 3) decomposition of PAA during exposure of chicken wings to the PAA solutions.

## MATERIALS AND METHODS

### Bacterial Strains and Inoculum Preparation

Three *Salmonella* serotypes, including nalidixic acid-resistant *Salmonella* Typhimurium (**ST**) obtained from the U.S. National Poultry Research Center, United States Department of Agriculture, Athens, GA; rifampicin-resistant strains of SI (5 isolates; SI) and kanamycin-resistant *Salmonella* Reading (**SR**) (5 isolates; SR) were used (Table 1). The wild-type SI and Reading were obtained from the USDA-FSIS and were isolated from chicken meat and turkey products, respectively. The wild-type SI and Reading were adapted to 200 ppm of rifampicin and kanamycin, respectively by serial transfers to increasing concentrations of respective antibiotics. As the objective of the study is to evaluate the differences in PAA resistance of the *Salmonella* serotypes, the PAA resistance of individual strains of each serotype and those of the wild and antibiotic-transformed serotypes and strains was not evaluated.

**Table 1:** Sources of *Salmonella* serotypes used for evaluation of resistance to PAA when inoculated on chicken wings.

Serotype		Isolate	Description
Typhimurium^1^		USDA ARS	Poultry (unknown)
Infantis^2^		FSIS12138670	Raw-Intact-Chicken
		FSIS22130923	Raw-Intact-Chicken
		FSIS22130925	Raw-Intact-Chicken
		FSIS22130947	Young Chicken Carcass Rinse
		FSIS32104927	Raw-Intact-Chicken
Reading^3^		FSIS12030472	Raw-Ground, Comminuted (Nonintact-Turkey)
		FSIS12035798	Raw-Ground, Comminuted (Nonintact-Turkey)
		FSIS12138569	Raw-Ground, Comminuted (Nonintact-Turkey)
		FSIS22029230	Raw-Ground, Comminuted (Nonintact-Turkey)
		FSIS22029469	Raw-Ground, Comminuted (Nonintact-Turkey)

1 *Salmonella* Typhimurium was obtained from Dr. Nelson Cox, USDA ARS, National Poultry Research Center, Athens, GA and the serotype was resistant to nalidixic acid (200 ppm).

2,3 *Salmonella* Infantis and Reading isolates were obtained from USDA FSIS Regional Laboratory, Athens, GA and trained on Rifampicin (200 ppm; Infantis) or Kanamycin (200 ppm; Reading) through serial transfers to increasing concentrations of the respective antimicrobial.

A loopful culture of ST, SI, and SR from frozen glycerol stocks (-80°C) was streaked onto Brilliant Green Sulfa agar (BGS; Difco**,** Sparks, MD) supplemented with 200 ppm of nalidixic acid (BG*^Nal^*; Sigma-Aldrich, St. Louis, MO), rifampicin (BG*^Rif^*; 200 ppm) or kanamycin (BG*^Kan^*; 200 ppm), respectively and incubated for 24 h at 35 ± 1°C. A cocktail of ST, SI and SR was used to inoculate chicken wing flats (skin-on). An isolated colony of ST from BG Sulfa agar was used to inoculate 50 mL of Tryptic Soy Broth (TSB; Remel, Lenexa, KS) supplemented with 200 ppm nalidixic acid and were incubated for 18 to 24 h at 35 ± 1°C. Similarly, isolated colonies obtained from 5 strains each of SI and SR were inoculated separately in 10 mL sterile tryptic soy broth (**TSB**) tubes and incubated at 35 ± 1°C for 18 to 24 h. After incubation, the bacterial cultures were centrifuged individually at 5,500 × *g* for 10 min at 4°C, and the pellet was then re-suspended with 15 mL 0.1% peptone water (PW; Difco™) and centrifuged following the same conditions as described. The supernatant was removed, and the pellet was re-suspended in 5 mL of PW, transferred to 150 mL of sterile PW and used for inoculation.

The final concentration of each bacterial suspension was ca. 6 log CFU/mL in 150 mL of PW as confirmed by direct plating. For each replication, a new inoculum was prepared on the day of the experiment.

### Preparation of Peroxyacetic Acid Solutions

Peracetic acid solutions (SaniDate FD Plus; BioSafe Systems, LLC, East Hartford, CT) were prepared by diluting appropriate PAA volume in prechilled water (4°C) to obtain final concentrations of 100 and 500 ppm and were either unadjusted (natural pH) or adjusted to pH 8.5 using 10 N NaOH (Mallinckrodt Baker Inc., Phillipsburg, NJ) diluted to 1 N in deionized water. PAA concentrations were confirmed using Peracetic Acid Test Kit (LaMotte Company, Ocean City, MD). Stabilized PAA (SaniDate FD Plus) is a commercially available PAA solution marketed as 19.5 to 25% peracetic acid, 9.5 to 12% hydrogen peroxide, 36 to 39% acetic acid and 1-hydroxyethylidene-1,1-diphosphonic acid (**HEDP**) <1% was obtained from the manufacturer and stored under refrigeration until use.

### Preparation of Antimicrobial Treatments

The antimicrobial treatments evaluated in this study included 100 and 500 ppm PAA at 2 pH levels (natural/unadjusted and 8.5). The pH of PAA solutions was adjusted to pH 8.5. The solutions were prepared in plastic buckets (18.9 L; Encore Plastics, Forsyth, GA) using pre-chilled water (≤4°C). The pH of each treatment was confirmed using a pH meter (Orion Star A111, Thermo Scientific, Ward Hill, MA). The buckets containing the PAA solutions were placed in baskets filled with ice to maintain the solution temperature (≤4–5°C).

### Inoculation of Chicken Wings

Fresh bone-in, skin-on broiler chicken wing flats (wings) were obtained from a local commercial poultry processing facility on each day of the experiment. For each PAA treatment, chicken wings (ca. 0.45 kg) were placed on a sterile stainless-steel rack and inoculated with the ST, SI, and SR cocktail by spraying 5 mL on each side and spread evenly using sterile tongs and placed in a laminar flow biological safety cabinet for 15 min to allow bacterial attachment.

### Application of Antimicrobial Treatments

The inoculated chicken wings were transferred to a sterilized stainless steel wire mesh basket (Model DND-095RND120-C04S, Any size basket, York, PA) and immersed in containers containing PAA solutions of appropriate concentration (100, 500 ppm), and pH (unadjusted [natural] or pH 8.5) for either 10 s or 60 min to replicate treatments applied to chicken parts and whole chicken carcasses, respectively. Compressed air (103.42 kPa) was pumped to provide agitation in the PAA solution using an air compressor (Model D55146 Air Compressor, Dewalt, Towson, MD) by the means of 2 concentric circular plastic tubes (tube ID 0.64 cm; dia. of the concentric tubes - 11.5 cm [inner], 15.4 cm [outer], with 1.6 mm holes drilled at 2.5 cm intervals; McMaster-Carr, Elmherst, IL) affixed to a concentric ceramic plate at the bottom of the buckets to facilitate uniform distribution of compressed air. Three independent replications were performed for each treatment on different days, with fresh inoculum and antimicrobial treatments on each day of the experiment. Inoculated wings not subjected to any antimicrobial treatment served as a control.

### Bacterial Enumeration

Chicken wings treated with PAA solutions were aseptically transferred into sterile rinse bags (BRB3500; 3M Food Safety, St. Paul, MN) and rinsed manually with 100 mL of chilled Buffered Peptone Water (BPW; Difco**,** Sparks, MD) supplemented with sodium thiosulfate (0.1%; Acros Organics, NJ) to neutralize the effects of residual PAA on the chicken wings for 1 min. Rinsates for each sample were collected and serially diluted in PW supplemented with 200 ppm nalidixic acid for ST, PW supplemented with rifampicin (200 ppm) and kanamycin (200 ppm) for *Salmonella* SI and SR enumeration, respectively*.* Appropriate serial dilutions were plated on Aerobic Plate Count Petrifilm (APC; 3M Food Safety, St. Paul, MN) and incubated at 35 ± 1°C for 24 h; enumerated and reported as log_10_ CFU/mL.

### Color Measurement

Chicken wings were assessed for changes in objective color values (CIE: L*a*b*) on control and treated product immediately after the application of treatments, using a Konica Minolta colorimeter (CR400, Konica Minolta Inc., Ramsey, NJ). The colorimeter was initially calibrated with a reflectance standard plate supplied by the manufacturer and the L* (lightness), a* (redness), and b* (yellowness) values were recorded by placing the hand-held colorimeter directly in contact with the skin. Triplicate measurements were obtained from 3 separate chicken wings and averaged for analysis.

### Decomposition of PAA

To determine the effect of pH levels on the decomposition of PAA, the PAA concentration was assessed after each immersion treatment. Similarly, to assess the stability of pH in PAA solutions after immersion of chicken wings, pH was noted after 10 s, 30 min and 60 min using a portable pH meter (Oakton pH Testr® 5F).

### Experimental Design and Statistical Analyses

A 3-*Salmonella* serotype (ST, SI and SR) x 2 PAA conc (100 and 500 ppm) x 2 pH values (unadjusted [natural] and pH 8.5) x 2 exposure times (10 s and 60 min) factorial design was used. Three independent replications were performed on different days using fresh chicken wings, PAA solutions and fresh bacterial inoculum. Data were analyzed using 3-way ANOVA (for dependent variables) in the GLM of SAS 9.4 (2004; SAS Institute Inc., Cary, NC). Fisher's least significant difference (α = 0.05) was used to separate means of the microbial reductions (log CFU/mL).

## RESULTS AND DISCUSSION

The reductions in *Salmonella* serotype (Typhimurium, Infantis and Reading) population after exposure to PAA solutions (various concentrations, pH and exposure time) on inoculated chicken wings and on skin color was evaluated. In addition, changes in pH of the PAA solutions and decomposition of PAA after exposure to the chicken wings was also evaluated. The mean pH values of PAA solutions were 3.05 ± 0.12 and 3.00 ± 0.05 for 100 and 500 ppm PAA concentrations, respectively. Similarly, Kumar et al. (2020) reported an average pH of 2.6 ± 0.3 and 2.8 ± 0.2 for PAA concentrations 100 and 500 ppm, respectively. These differences could be due to differences in the PAA:acetic acid ratio in the commercial PAA solutions supplied by the manufacturer. Typical PAA exposure times of 10 s or 60 min were used to simulate poultry parts antimicrobial treatment and the chiller treatment of broiler carcasses, respectively. Similarly, pH of antimicrobial treatments was either unadjusted or adjusted to pH 8.5 since most poultry processors prefer to adjust their PAA solutions towards alkaline range in the main chiller to enhance moisture gain by carcasses. Extended exposure (≥45 min) of carcasses to PAA at alkaline pH compared to acidic pH (natural pH) of commercially available PAA solutions (Vaddu et al., 2021a) in the chiller enhances moisture gain. The temperatures of the PAA solutions were maintained at 4 to 6°C throughout the study. However, for parts antimicrobial treatment, the pH was not adjusted as the treatment is for a short period and processors do not adjust the pH at this stage compared to during chilling of the broiler carcasses.

Peroxyacetic acid is the most popular poultry processing antimicrobial used in either pre-chill, chill, or post-chill interventions of whole carcasses or post-chill use for chicken parts. Several studies have reported the antimicrobial efficacy of PAA against ST, on whole broiler carcasses and parts. Nagel et al. (2013) reported 2.02 and 2.14 log CFU/mL reductions of inoculated (6 log CFU/mL) *S*. Typhimurium on broiler carcasses that were immersed for 20 s in a post-chill dip tank at PAA concentrations 400 and 1,000 ppm, respectively. In a subsequent study, immersion treatment of chicken wings in 500 ppm PAA for 10 s and 60 min lowered inoculated (6 log CFU/mL) *S*. Typhimurium by 1.0 and 2.1 log CFU/mL, respectively (Kataria et al., 2020). Limited studies have investigated the antimicrobial efficacy of PAA on *S*. Reading and *S*. Infantis on broilers or turkeys. Olson et al. (2020) investigated the antimicrobial efficacy of PAA against *S*. Reading on skin-on turkey drumsticks, wherein the authors reported that PAA (500 ppm) used as an immersion dip for 30 s reduced *S*. Reading by 2.6 log CFU/mL compared to the controls. Wythe et al. (2021) used immersion in PAA (0.06%, 20 s) to significantly reduce SI (1.14 log CFU/g) on skin-on chicken thighs. Increased concern among poultry processors on potentially enhanced resistance of *S*. Infantis and *S*. Reading to antimicrobial interventions used in poultry processing makes it necessary to determine the efficacy of PAA on the 3 *Salmonella* serovars (Typhimurium, Infantis and Reading).

Statistical analysis indicated that interactions were not observed for all combinations of treatments and hence, individual treatments (*Salmonella* serovars, PAA concentration, pH and immersion time) were evaluated, while combining all other parameters to evaluate the effect of these individual treatments and will be discussed (Table 2).

**Table 2:** Probability values for the main effects and interactions for reduction in *Salmonella* serotype (Ser; Typhimurium [**ST**], Infantis [**SI**] and Reading [**SR**]) on inoculated chicken wings immersed in peroxy acetic acid (**PAA**) solution of different concentrations (Conc; 100 and 500 ppm) and pH values (pH; natural and 8.5) for 10 s and 60 min.

Source	Pr > F
Conc	<0.0001
pH	0.8490
Conc*pH	0.1403
Time	<0.0001
Conc*Time	0.4243
pH*Time	0.9310
Conc*pH*Time	0.1641
Ser	0.0165
Conc*Ser	0.8331
pH*Ser	0.7944
Conc *pH*Ser	0.6146
Time*Ser	0.5930
Conc *Time* Ser	0.8186
pH*Time* Ser	0.7518
Conc *pH*Time*Ser	0.6111

### *Salmonella* Reductions (ST, SI, and SR)

The inoculation levels of 3 serovars of *Salmonella* (ST, SI, SR) on the chicken wings following the inoculation were 6.70 ± 0.12, 6.86 ± 0.13 and 6.82 ± 0.12 log CFU/mL, respectively of each serovar. Naturally occurring background microbial populations resistant to 200 ppm nalidixic acid, rifampicin or kanamycin were not detected (<1 CFU/mL) on the chicken wings used in the experiment.

**Figure 1:**
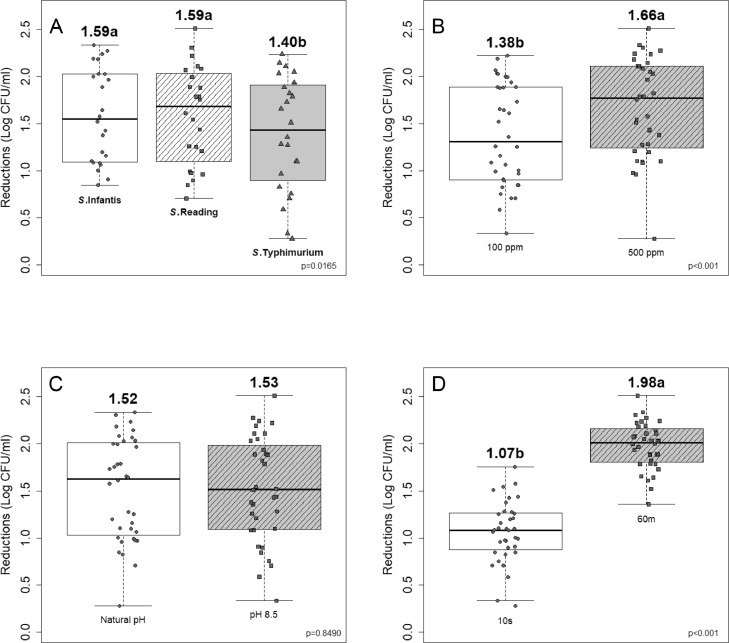
Boxplots of the effect of individual parameters (*Salmonella* serovars [A], PAA concentration [B], PAA pH [C] and time of exposure [D]) on *Salmonella* reductions. All possible interactions were non-significant (*P* > 0.05) and hence, each individual parameter was evaluated. Same alphabets above the boxes indicate the treatments were similar (*P* > 0.05); and the statistical significance values within the box plots indicates the level of significance for each individual parameter.

Immersion of inoculated chicken wings in PAA solutions, regardless of the concentration, pH and immersion time resulted in ST, Infantis and Reading reductions of 1.40, 1.59, and 1.59 log CFU/mL, respectively (*P* ≤ 0.05; Fig. 1). While the *Salmonella* population reductions were greater for SI and Reading (*P* ≤ 0.05) compared to Typhimurium, these differences (0.20 log CFU/mL) probably are not biologically significant. Immersion of inoculated chicken wings in unadjusted PAA solutions of 100 ppm for 10 s resulted in similar ST, SI, SR reductions of 0.83, 1.08, and 1.03 log CFU/mL, respectively (Table 3). Regardless of the PAA pH and concentration (100 ppm, 500 ppm), increased exposure time (60 min vs. 10 s) resulted in greater reduction (*P* ≤ 0.05) of ST, SI, and SR. The most effective treatment resulting in greater reductions of ST, SI, and SR was 500 ppm of PAA exposed for 60 min, resulting in approximately 2 log CFU/mL. Zhang et al. (2018) reported a 1.5 log CFU/mL reduction of ST on mixed chicken parts (1.85 kg including breasts, thighs, wings, and drumsticks) that were immersed in PAA (700 and 1,000 ppm) for 23 s. Microbial reductions increased numerically with increasing PAA concentrations (from 100–500 ppm) for 10 s and 60 min exposure (*P* > 0.05). This is consistent with the findings of Kataria et al. (2020) and Vaddu et al. (2021a) describing increasing PAA (from 50 to 500 ppm) and immersion for 10 s showed similar reductions of ST.

**Table 3:** Reduction in *Salmonella* serotype (Typhimurium, Infantis, and Reading) populations (Mean ± SD; log CFU/mL) on inoculated chicken wings after exposure to different concentrations (100 and 500 ppm) of peroxyacetic acid [**PAA**] at pH levels (unadjusted [natural] or pH 8.5) for either 10 s or 60 min.

Conc. (ppm)	Exposure Time	*Salmonella* Typhimurium		*Salmonella* Infantis		*Salmonella* Reading	
		Natural pH	pH 8.5	Natural pH	pH 8.5	Natural pH	pH 8.5
100	10 s	0.83 ± 0.13^abx^	0.56 ± 0.21^ax^	1.08 ± 0.08^bx^	0.95 ± 0.12^abx^	1.03±0.21^bx^	0.95±0.28^abx^
	60 min	1.81 ± 0.20^ay^	1.73 ± 032^ay^	1.89 ± 0.21^ay^	1.87 ± 0.34^ay^	1.89±0.21^ay^	2.00±0.19^ay^
500	10 s	0.89 ± 0.53^ax^	1.30 ± 0.19^abz^	1.29 ± 0.25^abx^	1.30 ± 0.19^abx^	1.23±0.45^abx^	1.40±0.17^bz^
	60 min	2.06 ± 0.24^ay^	2.00 ± 0.15^ay^	2.16 ± 0.19^ay^	2.18 ± 0.13^ay^	2.06±0.26^ay^	2.14±0.36^ay^

Same superscripts (^ab^) within the same row indicate no significant differences (*P* > 0.05); Same superscripts (^xyz^) within the same column indicate no significant differences (*P* > 0.05).

Regardless of the other parameters, increasing the PAA concentration (100 vs. 500 ppm) resulted in greater reduction of *Salmonella* population (1.38 vs. 1.66 log CFU/mL; *P* ≤ 0.05); increasing the exposure time (10 s vs. 60 min) resulted in greater reduction of *Salmonella* population (1.07 vs. 1.98 log CFU/mL; *P* ≤ 0.05), whereas adjusting the pH of the PAA solution to pH 8.5 did not affect the *Salmonella* reduction (1.52 vs. 1.53 log CFU/mL).

### Skin Color

Poultry skin color can be affected by antimicrobials which rely on low pH for antimicrobial activity or oxidizers such as chlorine and PAA and the degree of discoloration depends on the concentration, exposure time and temperature (Vaddu et al., 2021b). Bleaching of chicken skin can lead to a lighter color making it appear lighter. Most consumers perceive “golden yellow’’ chicken skin color as quality and freshness of meat. Further, processors consider adjusting pH to higher values (alkaline range, pH >8.5) to aid in moisture gain, while exposure to acidic pH results in moisture loss and reduction in carcass yield. Some commercial poultry processors use soft scalding method to preserve the cuticle and hence the yellow color to meet the consumer demand. Poultry processors, especially those that market whole carcasses consider preserving the skin color essential after PAA treatments. Immersion of chicken wings in PAA solutions, regardless of the concentration, pH level, and exposure time did not affect (*P* > 0.05) the L* values compared to the non-treated control (Table 4) except for the chicken wings immersed in non-adjusted PAA solution (natural). Immersion in PAA solution of higher concentration (500 ppm) for 60 min showed higher L* value compared to controls (*P* ≤ 0.05). Likewise, no differences in a* and b* values were observed between the treatments (*P* > 0.05), regardless of the pH, concentration, and exposure time. Similarly, Scott et al. (2015) and Vaddu et al. (2021b)reported no differences in color of skin on chicken wings when immersed in 100 and 500 ppm PAA solutions for 60 min and 20 s respectively. Bauermeister et al. (2008) reported no significant differences in color when broiler carcasses were immersed in PAA (200 ppm) for 60 min, but carcasses immersed in 30 ppm chlorine had lower L* values (*P* ≤ 0.05).

**Table 4:** Instrumental color (L*, a*, and b*) of chicken wings after exposure to different concentrations (100 and 500 ppm) of peroxyacetic acid at pH levels (unadjusted [natural] or pH 8.5) for either 10 s or 60 min.

		L*		a*		b*	
Conc. (ppm)	Exposure time	Natural pH	pH 8.5	Natural pH	pH 8.5	Natural pH	pH 8.5
Control	None	78.72 ± 0.64^ax^	-	1.57 ± 1.11^ax^	-	1.42 ± 0.66^ax^	-
100	10 s	78.45 ± 0.53^ax^	79.90 ± 0.56^ax^	0.02 ± 1.18ax	0.57 ± 2.91^ax^	1.23 ± 1.30^ax^	3.63 ± 0.45^ax^
	60 min	80.18 ± 0.94^axy^	79.67 ± 1.34^ax^	0.60 ± 0.99ax	0.33 ± 0.97^ax^	0.56 ± 2.67^ax^	2.62 ± 0.99 ^ax^
500	10 s	78.71 ± 0.92^ax^	79.02 ± 0.63^ax^	0.66 ± 1.87ax	0.56 ± 1.31^ax^	2.99 ± 0.91^ax^	1.39 ± 0.91^ax^
	60 min	80.97 ± 0.49^ay^	80.46 ± 0.61^ax^	-0.76 ± 0.45ax	-0.30 ± 1.33^ax^	1.48 ± 2.45^ax^	1.64 ± 2.01 ^ax^

Same superscripts (^ab^) within the same row indicate no significant differences (*P* > 0.05); Same superscripts (^xyz^) within the same column indicate no significant differences (*P* > 0.05).

A potential pitfall in this study in terms of color of chicken wings was that chicken wings were obtained from a commercial processor that uses hard scalding (higher temperatures) after traditional immersion chilling without PAA treatment and does not supplement color generating pigments in feed while rearing broilers. Further, the processor does not market whole birds and thus, does not focus on the broiler skin color for marketing. Thus, the lack of discoloration during immersion in PAA could be due to either hard scalding or from birds not showing significant pigmentation.

### Decomposition of PAA

PAA has emerged as a predominant antimicrobial/processing aid in poultry processing to reduce the populations and/or prevalence of *Salmonella* and *Campylobacter* in poultry. Several studies have reported the application of PAA in poultry processing related to reductions in foodborne pathogens during exposure. The pH of PAA solutions after different immersion treatments increased from 3.0 and 3.9 in unadjusted [natural] pH treatment (100 ppm), while the pH declined subsequent to immersion of chicken wings from 8.5 and 7.1 in pH 8.5 adjusted PAA solution (100 ppm). Yuan et al. (1997) investigated the effect of pH on PAA decomposition in aqueous solutions. They reported higher consumption rate of PAA at higher pH levels ≥ 8.2 due to higher spontaneous decomposition rates and hydrolysis at higher pH levels. Similarly, we observed greater decomposition of PAA (Table 5) at higher pH value and at longer times (10 s vs. 60 min). At pH values greater than pK_a_ of PAA (8.2), disassociated acid form would be predominant resulting in higher PAA decomposition (Kitis, 2004). However, no significant differences in efficacy of PAA (50 and 500 ppm) in reducing *Salmonella* and *Campylobacter* on inoculated chicken wings at various pH levels were observed by Kataria et al. (2020) and Vaddu et al. (2021a). After immersion treatments, the water treatment solutions were plated on APC Petrifilm with either nalidixic acid (200 ppm), rifampicin (200 ppm) or kanamycin (200 ppm) to observe any *Salmonella* serovars (ST, SI, SR) rinsed off the chicken wings after immersion treatments. No viable population were observed in the treated antimicrobial solutions. This also implies the risk of cross contamination between the parts or in case of the whole birds within the whole bird chiller is minimal.

**Table 5:** PAA solution pH and concentration before and subsequent to immersion of *Salmonella*-inoculated chicken wings for various time periods.

		Solution pH			Concentration (ppm)		
Target PAA Conc.	Target pH	Before^1^	10 s	60 min	Before	10 s	60 min
100 ppm	Nat.	3.50 ± 0.12^a^^2^	3.64 ± 0.07^a^	3.85 ± 0.13^b^	110 ± 9^a^	105 ± 0^ab^	95 ± 9^b^
	8.50	8.50 ± 0.00^a^	7.36 ± 0.09^b^	7.18 ± 0.24^b^	110 ± 9^a^	80 ± 9^b^	60 ± 0^c^
500 ppm	Nat.	3.00 ± 0.05^a^	3.05 ± 0.03^ab^	3.15 ± 0.09^b^	510 ± 0^x^	455 ± 17^b^	400 ± 23^c^
	8.50	8.48 ± 0.02^a^	7.66 ± 0.31^b^	7.07 ± 0.31^c^	505 ± 9^x^	315 ± 40^b^	240 ± 26^c^

1 Before: PAA solution pH and concentrations after preparation of the solutions and prior to immersion of the chicken wings; 10 s and 60 min indicate the values after immersion of the chicken wings for specific period of time; Nat.: Natural, non-adjusted pH of the PAA solution.

2 Same superscripts within each concentration and target pH indicate the values are similar (*P* > 0.05) within the solution pH and solution concentration.

## CONCLUSIONS

Immersion of inoculated chicken wings in PAA solutions (100 or 500 ppm) were effective (*P* < 0.05) in reducing different *Salmonella* serovars (Typhimurium, Infantis, and Reading). Irrespective of the PAA concentrations and pH levels, greater microbial reductions were observed at longer exposure times (10s vs. 60 min). Susceptibility to PAA varied among the serotypes. *S*. Typhimurium was found to be relatively more resistant to PAA solutions than *S*. Infantis and *S*. Reading, regardless of the experimental conditions (PAA concentration [100 or 500 ppm]; pH values [natural/unadjusted pH or 8.5 pH]; and exposure time [10 s or 60 min]). There is a need to assess the distribution of major *Salmonella* serovars in poultry among different geographical regions and evaluate the efficacy of different processing aids predominantly used in poultry production and processing. Minimal changes in color of chicken wings were observed upon immersion in PAA solutions. Rapid decomposition of PAA was observed in solutions adjusted to higher pH (8.5) than in solutions left unadjusted.

## DISCLOSURES

The authors declare no conflicts of interest.
